# Inherited Platelet Disorders During Pregnancy and Delivery: Overview of Management Strategies and Emerging Therapeutic Considerations

**DOI:** 10.3390/hematolrep18020016

**Published:** 2026-02-26

**Authors:** Victor Zibara, Nicoletta Machin

**Affiliations:** 1 Division of Classical Hematology, Department of Medicine, University of Pittsburgh Medical Center, Pittsburgh, PA 15219, USA; 2 Hemophilia Center of Western Pennsylvania, Pittsburgh, PA 15213, USA

**Keywords:** inherited platelet disorders, pregnancy, delivery, women

## Abstract

Inherited platelet disorders (IPDs) comprise a heterogeneous group of rare conditions that present particular challenges during pregnancy, with bleeding risk increasing during labor and the immediate postpartum period. These disorders require coordinated, multidisciplinary management to mitigate maternal and neonatal bleeding risk. Although data remains limited, individuals with IPD, including Bernard–Soulier syndrome, Glanzmann thrombasthenia, MYH9-related disorders, Hermansky–Pudlak syndrome, and platelet storage pool disorders, are at an increased risk for obstetrical bleeding, with the degree of risk varying by underlying diagnosis. In severe inherited platelet disorders such as Glanzmann thrombasthenia, peripartum hemorrhage is common, with up to half of the deliveries in some series requiring red cell or platelet transfusion. Because these conditions are congenital, the fetus may also be affected, placing neonates at risk for serious bleeding complications, including intracranial hemorrhage, although available data is limited. Despite the considerable morbidity and mortality risk associated with inherited platelet disorders, management strategies during pregnancy and delivery remain poorly defined. This stands in contrast to other bleeding disorders, such as factor deficiencies, for which multiple therapeutic approaches have been evaluated in the peripartum setting. In this review, we summarize the available evidence and current management strategies for individuals with inherited platelet disorders during pregnancy and delivery.

## 1. Introduction

Inherited platelet disorders (IPDs) are a heterogeneous group of bleeding diatheses associated with defects in platelet function or platelet number, comprising inherited platelet function disorders and inherited platelet number disorders [1]. Inherited platelet function disorders are characterized by quantitative or functional abnormalities in platelet surface receptors, signaling pathways, or intracellular processes required for normal platelet activation, while inherited platelet number disorders are distinguished primarily by thrombocytopenia and are often accompanied by extra-hematologic manifestations [1]. IPDs also encompass rare disorders affecting the platelet membrane phospholipid system, such as Scott syndrome, as well as uncommon inherited thrombocytopenias, although pregnancy-related clinical data for these entities remain extremely limited.

**Figure 1:**
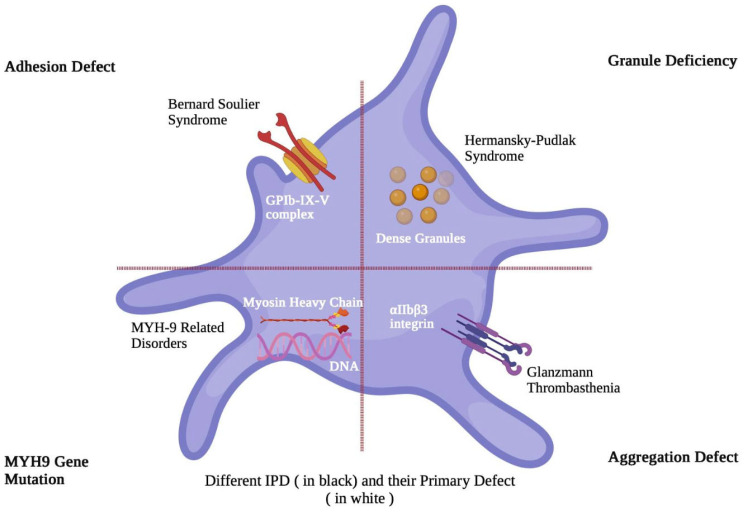
Inherited platelet disorders.

These conditions include Bernard–Soulier syndrome, Glanzmann thrombasthenia, MYH9-related disorders, Hermansky–Pudlak syndrome, and platelet storage pool disorders, all of which demonstrate wide genotypic and phenotypic heterogeneity (Figure 1). Most IPDs affect both men and women and are associated with variable bleeding phenotypes, typically presenting as mucocutaneous bleeding, but may also manifest as bleeding following trauma or minor surgical procedures, and in some cases, life-threatening central nervous system hemorrhage. In women, menorrhagia and abnormal uterine bleeding, as well as delivery complications, including postpartum hemorrhage, are common clinical manifestations [2]. Even mild inherited platelet disorders can significantly impact quality of life in women and are associated with substantial morbidity, particularly through menstrual bleeding complications such as iron deficiency anemia and pregnancy-related bleeding events [3].

Pregnancy and delivery represent a major hemostatic challenge for all women. In women with IPDs, the baseline risk of postpartum hemorrhage is further increased, and neonatal bleeding risk is also heightened [4]. The challenges faced by pregnant women with underlying IPDs are unique, reflecting the combined hemostatic demands of labor and delivery, the risks of antepartum and postpartum hemorrhage, and anesthesia-related complications.

Different therapeutic options have been used in the management of pregnancy-related bleeding in patients with underlying inherited platelet disorders. Despite the availability of multiple hemostatic agents, functional platelet testing during pregnancy is not currently a practical option to guide therapeutic decision making, particularly in the peripartum setting. Desmopressin (DDAVP), a synthetic analog of the natural antidiuretic hormone L-arginine vasopressin, has been shown to improve hemostasis through activation of endothelial V2 vasopressin receptors and cyclic adenosine monophosphate (cAMP)-dependent signaling, leading to the release of von Willebrand factor and tissue plasminogen activator from Weibel–Palade bodies [5]. Another widely used option is tranexamic acid (TXA), an antifibrinolytic agent that inhibits plasminogen activation and fibrinolysis, thereby stabilizing the fibrin clot formed by the coagulation cascade [6]. Recombinant activated factor VII (rFVIIa) also plays a role in controlling bleeding as a bypassing agent by directly activating factor X on the surface of activated platelets, thereby promoting thrombin generation and fibrin clot formation independently of factor VIII and factor IX [7]. This mechanism partially compensates for platelet quantitative or functional defects by enhancing thrombin generation on activated platelet surfaces, strengthening clot formation in settings where the normal thrombin burst is impaired [8]. Finally, platelet transfusion may be used in selected situations when platelet refractoriness or alloimmunization is not a concern.

**Figure 2:**
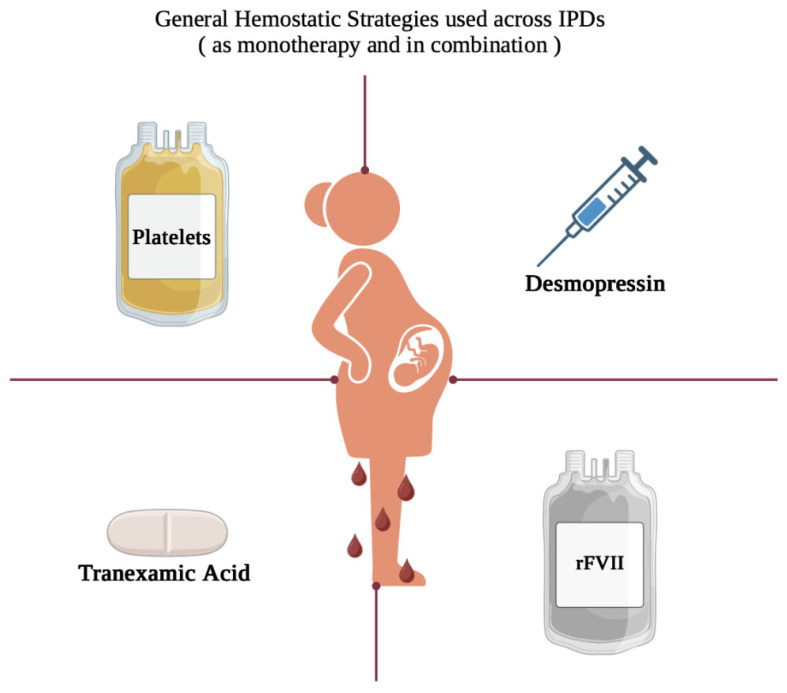
Mainstay of therapy in patient with underlying IPDs. rFVII: Recombinant Factor VII.

Despite these challenges, evidence-based guidelines for the management of IPDs in pregnancy remain limited, largely due to disease rarity, with most recommendations derived from case series and expert consensus. This review synthesizes current knowledge on the management of pregnancy and delivery in women with IPDs, highlighting available therapeutic options (Figure 2) and best practices for intrapartum and postpartum care.

A literature search was performed using PubMed and Embase to identify relevant studies. The search strategy incorporated a combination of keywords and MeSH terms, including “pregnancy,” “delivery,” “bleeding,” and “inherited platelet disorders.” Articles were limited to those published in English between 1990 and 2025. Retrieved records were screened by title and abstract, with full-text articles reviewed for relevance to pregnancy and peripartum management in inherited platelet disorders. Given the rarity of these conditions and the ethical and practical challenges of conducting interventional studies in pregnant populations, the majority of evidence summarized in this review is derived from case series, observational studies, and expert opinion, unless otherwise specified in the relevant sections.

## 2. Glanzmann Thrombasthenia


**Key Points: Glanzmann Thrombasthenia**

GT is a severe inherited platelet function disorder with a high risk of hemorrhage during delivery and postpartum, despite supportive therapy.Miscarriage and antepartum bleeding are not increased, but invasive procedures (amniocentesis, CVS, D&C) carry an elevated bleeding risk.Postpartum hemorrhage occurs in >50% of pregnancies and is the highest-risk period.Platelet transfusions should be avoided unless necessary due to risk of HLA or αIIbβ3 alloimmunization and future refractoriness.Recombinant activated factor VII (rFVIIa) is a key hemostatic agent, particularly in alloimmunized patients or when platelets are ineffective.Delivery should occur at a specialized center with multidisciplinary planning and access to matched platelets, rFVIIa, TXA, and neonatal support.Avoid vacuum-assisted delivery; forceps may be considered only if urgently required.Neonatal risk includes inherited GT and alloimmune thrombocytopenia; early pediatric involvement and monitoring are essential.


### 2.1. Overview and Pathophysiology

Glanzmann thrombasthenia (GT) is a rare inherited platelet disorder, caused by a deficiency or dysfunction of the platelet αIIbβ3 integrin, resulting in impaired platelet aggregation and subsequent failure of primary hemostasis [9]. Clinically, GT presents with mucocutaneous bleeding of variable severity, ranging from minor epistaxis or gingival bleeding to spontaneous or trauma-associated hemorrhage [10].

Although rare globally, GT is one of the most clinically significant inherited platelet disorders in reproductive-aged women due to the severity of bleeding phenotype and high postpartum hemorrhage risk. The 2023 World Federation of Hemophilia registry identified 1917 females living with GT worldwide, accounting for approximately 5% of bleeding disorders reported in women [11].

### 2.2. Pregnancy-Associated Bleeding Risk

Pregnancy in individuals with GT is associated with a high risk of hemorrhage, particularly during delivery and the postpartum period. Rates of postpartum hemorrhage (PPH) as high as 58–64% have been reported despite use of hemostatic therapy [4,12,13]. In contrast, available data do not suggest increased rates of miscarriage or antepartum hemorrhage; however, bleeding risk increases substantially in the context of invasive procedures or obstetric complications. Importantly, pregnancy does not improve platelet function in GT, and bleeding risk persists throughout gestation.

Because of the high hemorrhagic risk, individuals with GT should receive care at centers with multidisciplinary expertise, including hematology, obstetrics, anesthesia, transfusion medicine, pharmacy, genetics, and neonatology [14].

### 2.3. Antepartum Considerations

Minor bleeding during pregnancy is generally managed with local measures and antifibrinolytics [15]. Heavy menstrual bleeding is common prior to pregnancy, and preconception optimization, including correction of iron deficiency, is recommended to reduce baseline morbidity. Platelet transfusion is reserved for life-threatening bleeding because of the risk of alloimmunization against HLA class I and/or αIIbβ3 antigens. Recombinant activated factor VII (rFVIIa) has been used as an alternative in the presence of alloantibodies or when platelet transfusion is ineffective [12].

When invasive procedures such as amniocentesis or chorionic villus sampling are necessary, bleeding risk is increased. Although standardized treatment strategies are lacking, case-based practice supports the use of rFVIIa at 90 mcg/kg immediately prior to the procedure and every two hours post procedure for up to 12 h, in combination with antifibrinolytics [16].

### 2.4. Alloimmunization and Maternal–Fetal Impact

Alloimmunization occurs in approximately 20–30% of individuals with GT [17,18]. Anti-αIIbβ3 antibodies may contribute to treatment resistance, acute transfusion reactions, and fetal/neonatal complications including intracranial hemorrhage (ICH) [19,20,21,22].

Monitoring strategies typically mirror those used in other alloimmune neonatal platelet disorders. Individuals with

- **Prior pregnancy complicated by neonatal ICH** should undergo early intervention beginning at 12–16 weeks of gestation.
- **High or rising αIIbβ3 antibody titers** may require intervention beginning at 20–22 weeks.
- **Low or stable titers** are monitored routinely.
- **No history of alloimmunization** should undergo screening in the third trimester [22].

**Figure 3:**
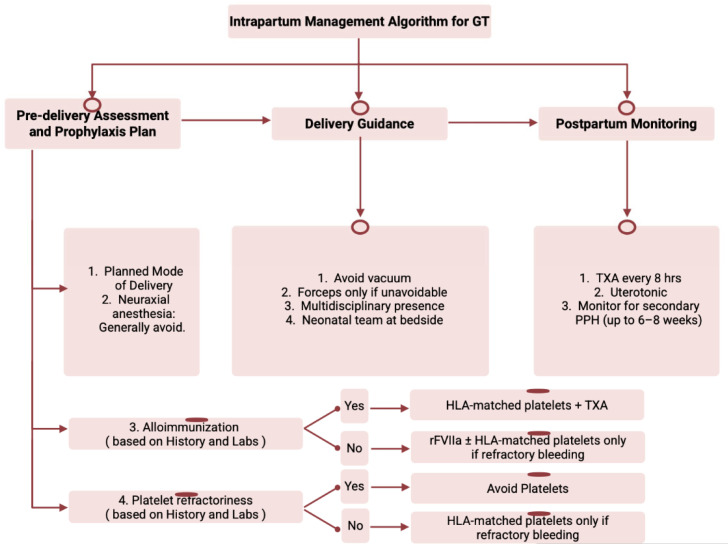
Intrapartum management algorithm for GT. GT: Glanzmann thrombasthenia; TXA: tranexamic acid; PPH: postpartum hemorrhage; rFVIIa: Recombinant Factor VII.

Therapies used to reduce antibody burden have included corticosteroids, IVIG, and rarely plasma exchange [19,20,21]. In a cohort of 40 pregnancies from 35 women with GT, two neonatal deaths due to ICH occurred in pregnancies where maternal anti-αIIbβ3 antibodies were present, emphasizing the need for prenatal antibody monitoring and counseling (Figure 3) [12,14].

### 2.5. Delivery Planning

Delivery should occur in a well-resourced referral center with access to matched platelets, rFVIIa, antifibrinolytics, and neonatal support. The choice of delivery mode (vaginal vs. cesarean) does not appear to influence the risk of PPH; however, a personal history of significant bleeding is a stronger predictor of hemorrhage risk [23,24,25].

Instrumental delivery should generally be avoided due to neonatal bleeding risk. Forceps may be considered only if urgently required; vacuum-assisted delivery is contraindicated. Neuraxial anesthesia is generally avoided unless hemostasis can be reliably achieved with rFVIIa or platelet support, given the risk of spinal or epidural hematoma (Figure 3) [23,24,25].

### 2.6. Intrapartum and Postpartum Management

Routine postpartum care includes standard uterotonics and administration of tranexamic acid at delivery, followed by continued dosing every eight hours. Patients require close surveillance for secondary PPH, which may occur up to 6–8 weeks postpartum.

Management strategies depend on alloimmunization status:

- **No alloimmunization or platelet refractoriness:** Platelet transfusion at least one hour prior to delivery may be considered. If bleeding occurs, HLA-matched platelets or rFVIIa may be used.
- **Presence of anti-HLA or anti-αIIbβ3 antibodies, or transfusion refractoriness:** rFVIIa (80–120 mcg/kg every 2–3 h after delivery) may be used as a primary strategy. Higher doses and adjunctive platelet transfusion may be necessary if bleeding persists [26].

In all cases, continued tranexamic acid and uterotonics are recommended (Figure 3) [12,13,27].

### 2.7. Neonatal Considerations

Because neonates may inherit GT or experience immune-mediated thrombocytopenia, pediatric evaluation at delivery is recommended. Risk is highest in the setting of maternal alloimmunization. Routine avoidance of vacuum extraction is essential due to risk of neonatal ICH.

### 2.8. Evidence Gaps

Most available data derive from case series and expert consensus rather than controlled trials. Standardized approaches to maternal antibody monitoring, delivery prophylaxis, and neonatal management are needed. Prospective registries and harmonized reporting of obstetric outcomes would substantially strengthen the evidence base.

## 3. Bernard–Soulier Syndrome


**Key Points: Bernard–Soulier Syndrome**

BBS is a rare inherited platelet adhesion disorder characterized by thrombocytopenia and an increased risk of postpartum hemorrhage.Platelet transfusion remains an important hemostatic strategy, while recombinant activated factor VII may be considered in selected cases, particularly in the setting of platelet refractoriness or alloimmunization risk.Tranexamic acid is commonly used as adjunctive or prophylactic therapy during delivery and the postpartum period.Delivery should occur at a specialized center with multidisciplinary planning and access to transfusion support.Regional anesthesia is generally contraindicated due to the risk of spinal or epidural hematoma.


### 3.1. Overview and Pathophysiology

Bernard–Soulier syndrome (BSS) is an autosomal recessive inherited platelet disorder characterized by a defect of the GPIb–IX–V complex, an essential receptor for initiating primary hemostasis through interaction with von Willebrand factor. The syndrome was first described in 1948 by Bernard and Soulier in a young patient with thrombocytopenia, large platelets, and prolonged bleeding time [28].

BSS is extremely rare, with a prevalence of less than 1 in one million among European, North American, and Japanese populations. The most common clinical manifestations include epistaxis and gingival or cutaneous bleeding; however, menorrhagia is a frequent and often prominent symptom in menstruating women [28]. The management of BSS during pregnancy and delivery is particularly challenging due to the combined effects of thrombocytopenia and platelet dysfunction.

In contrast to Glanzmann thrombasthenia, bleeding risk in Bernard–Soulier syndrome during pregnancy and delivery is driven primarily by thrombocytopenia and impaired platelet adhesion, with platelet transfusion playing a central role in management.

### 3.2. Antepartum Considerations

Antenatal complications have been reported in approximately two-thirds of pregnancies in women with BSS, with severe thrombocytopenia being the most common complication, occurring in nearly half of reported cases. Hemorrhage and epistaxis have also been described, although less frequently.

### 3.3. Delivery Planning

As with Glanzmann thrombasthenia, women with BSS should undergo planned delivery at a tertiary care center with coordinated involvement of obstetrics, hematology, neonatology, anesthesia, and transfusion medicine. This is particularly important given the high risk of peripartum bleeding and the need for timely access to blood products.

A recent article by Saridogan et al. reviewed the literature on pregnancies in BSS patients. The authors noted a total of 51 pregnancies in 37 pregnant women in the review. PPH was observed in 52.9% of cases, with late PPH being more prevalent than early PPH at 35.3% vs. 31.4%, respectively. Interestingly, patients who received prophylactic platelet transfusions experienced less PPH. Also, it was noted that almost half of the pregnancies had severe thrombocytopenia with platelet counts <50,000/mm^3^ [29].

Cesarean delivery was the most common mode of delivery. Although postpartum hemorrhage appeared more frequent following vaginal delivery, these cases were also less likely to have received prophylactic therapy, limiting conclusions regarding the impact of delivery mode alone [29].

Regional anesthesia is generally contraindicated in women with BSS due to the risk of spinal or epidural hematoma. For cesarean delivery, general anesthesia is recommended, and the use of dibucaine as an anesthetic agent should be avoided [30].

### 3.4. Intrapartum and Postpartum Management

In terms of management for delivery, the recommendations for uncomplicated vaginal delivery include:

- rFVIIa at 90 mcg/kg every 2 h, for 3–4 doses or until adequate hemostasis;
- Tranexamic acid use.

As for caesarean sections or if bleeding occurs during vaginal delivery, the following are recommended:

- HLA-matched platelets;
- Tranexamic acid [30,31].

Prophylactic platelet transfusion should be used cautiously due to the risk of alloimmunization, which could increase the incidence of fetal neonatal alloimmune thrombocytopenia, which increases the risk for neonatal intracranial bleeding [32]. To reduce the risk of alloimmunization, HLA-matched platelets are hence recommended. The use of Intravenous Immunoglobulins (IVIGs), plasmapheresis and steroids has also been reported to enhance the response to platelet transfusions and prevent alloimmune neonatal thrombocytopenia [33,34].

FVIIa can be valuable especially in cases with platelet refractoriness; however, the risk of thromboembolism should be evaluated especially in cesarian sections received rFVIIa [29,32].

Tranexamic acid has been used prophylactically during delivery and is generally continued for at least two weeks postpartum due to the risk of late postpartum hemorrhage [29,32]. Aggressive use of uterotonic agents during the third stage of labor and avoidance of difficult instrumental deliveries are also recommended. Close postpartum monitoring for up to six weeks is advised.

Other Inherited Platelet Disorders:

## 4. Hermansky–Pudlak Syndrome


**Key Points: Hermansky–Pudlak Syndrome**

Hermansky–Pudlak syndrome is a rare autosomal recessive platelet delta storage pool disorder associated with systemic manifestations.Available data suggest low rates of antenatal bleeding complications in pregnancy, although published experience remains limited.Data on the guiding mode of delivery is sparse. Regional anesthesia is generally avoided, and instrumental delivery is typically minimized due to bleeding risk.Hemostatic management during delivery commonly includes desmopressin and or platelet transfusion, recognizing variable responses and limited evidence.


### 4.1. Overview and Pathophysiology

Hermansky–Pudlak syndrome (HPS) is a platelet delta storage pool disorder characterized by dense granule deficiency and is associated with oculocutaneous albinism. In addition to a bleeding diathesis, HPS is associated with a constellation of systemic manifestations, including visual impairment and, in some subtypes, renal and pulmonary involvement [35].

HPS is an autosomal recessive disorder, and affected individuals may present with bleeding symptoms such as epistaxis, excessive bleeding following procedures or surgery, heavy menstrual bleeding, and postpartum hemorrhage. These manifestations result from impaired platelet aggregation due to dense granule deficiency. HPS is most commonly reported in individuals from northwest Puerto Rico and the Swiss Valais region but is otherwise considered a rare disorder [36].

### 4.2. Pregnancy and Delivery-Associated Bleeding Risk

A study by Obeng-Tuudah et al. examined pregnancy and delivery outcomes in women with Hermansky–Pudlak syndrome. Among 29 reported pregnancies, 27 resulted in viable births. No antenatal bleeding events were reported, and no other pregnancy-related complications were noted [36].

In the same study, most deliveries were vaginal, and regional anesthesia was avoided in 92% of cases due to the underlying diagnosis. General anesthesia was used for all cesarean deliveries. Postpartum hemorrhage was reported in 44% of pregnancies, occurring in 53% of vaginal deliveries and 50% of cesarean deliveries [36].

### 4.3. Delivery Planning

Given the limited available data, there are no general recommendations regarding mode of delivery, and management should be individualized based on clinical presentation and bleeding risk. Similar to other inherited platelet disorders, instrumental delivery is generally avoided, although specific data for Hermansky–Pudlak syndrome are lacking [37]. The Royal College of Obstetricians and Gynecologists recommends avoiding regional anesthesia in patients with inherited bleeding disorders to reduce the risk of spinal or epidural hematoma and associated neurological complications [38].

### 4.4. Intrapartum and Postpartum Management

Among pregnancies with a known diagnosis of Hermansky–Pudlak syndrome at the time of labor, prophylactic hemostatic agents were used in 85% of cases, with 53% receiving desmopressin and 47% receiving platelet transfusions. Notably, cases managed with platelet transfusion demonstrated lower estimated blood loss and lower rates of postpartum hemorrhage compared with those treated with desmopressin alone. This finding is consistent with prior reports demonstrating variable hemostatic responses to desmopressin in platelet storage pool disorders [39].

Desmopressin can be used safely during pregnancy when clinically indicated. Considerations related to platelet transfusion mirror those in Glanzmann thrombasthenia and Bernard–Soulier syndrome, including the risk of alloimmunization and potential neonatal intracranial hemorrhage.

## 5. MYH-9-Related Disorders


**Key Points: MYH-9-Related Disorders**

MYH9-related disorders are a group of autosomal dominant conditions characterized by inherited thrombocytopenia and platelet macrocytosis due to variants in the *MYH9* gene.Thrombocytopenia represents the primary antepartum concern and requires close monitoring during pregnancy.Patients are at increased risk of bleeding, including postpartum hemorrhage, although reported risk varies across studies.Management during pregnancy and delivery is primarily supportive, with platelet transfusion used when clinically indicated; thrombopoietin receptor agonists remain investigational and have not been studied in pregnancy.


### 5.1. Overview and Pathophysiology

MYH9-related disorders (MYH9-RDs) are a group of autosomal dominant conditions characterized by inherited thrombocytopenia and platelet macrocytosis resulting from variants in the *MYH9* gene, which encodes the heavy chain of non-muscle myosin IIA. In addition to hematologic manifestations, MYH9-RDs may be associated with extra-hematologic involvement affecting multiple organ systems. MYH9-RDs were previously considered four distinct disorders with overlapping features, including May–Hegglin anomaly, Epstein syndrome, Fechtner syndrome, and Sebastian syndrome [40]. MYH9-RDs are rare; however, their true prevalence is likely underestimated due to underdiagnosis or misdiagnosis.

### 5.2. Antepartum Considerations

Women with MYH9-RDs are at increased risk of bleeding during menstruation, pregnancy, and delivery, similar to other causes of inherited thrombocytopenia, with overall risk higher than that of the general population. During pregnancy, patients are monitored closely with serial blood counts to assess platelet levels. Treatment is generally reserved for patients with platelet counts below 20 × 10^9^/L or those with active bleeding [41].

### 5.3. Delivery Planning

There are no specific recommendations regarding mode of delivery for women with MYH9-RDs. In a study by Noris et al., two neonates born to mothers with severe thrombocytopenia due to MYH9-RDs, who were delivered vaginally, died from intracranial hemorrhage. However, given the small number of events, it cannot be concluded that vaginal delivery is associated with an increased risk of neonatal intracranial hemorrhage [42].

### 5.4. Intrapartum and Postpartum Management

A retrospective study evaluating perioperative bleeding risk in patients with inherited platelet disorders included 829 surgical procedures, of which 75 involved patients with MYH9-RD. Patients who received prophylactic treatment prior to intervention demonstrated lower bleeding rates, including those with MYH9-RD who were managed with platelet transfusions and antifibrinolytic agents [43].

For delivery, platelet transfusion is often considered to achieve platelet counts of at least 70 × 10^9^/L when neuraxial anesthesia is anticipated [41]. Patients receiving transfusion should be monitored for transfusion reactions and alloimmunization. There are no reports specifically addressing desmopressin use during delivery in MYH9-RD; however, limited case reports in surgical settings suggest that prophylactic desmopressin may reduce bleeding risk [43].

Case series examining pregnancy outcomes in women with MYH9-RD have not demonstrated an increased risk of antepartum bleeding. Postpartum hemorrhage has been reported, although rates do not appear to exceed those of the general population [44].

### 5.5. Additional Potential Management Strategies

Thrombopoietin receptor agonists have been investigated in MYH9-RD, particularly in the perioperative setting for patients with severe thrombocytopenia. A phase II clinical trial demonstrated that Eltrombopag effectively increased platelet counts, with 78 percent of patients achieving platelet counts above 100 × 10^9^/L. However, pregnant women were not included in these studies [45,46]. Romiplostim has also been reported in perioperative case series, but its use has not been studied in pregnancy or during delivery [47].

## 6. Inherited Thrombocytopenias

Inherited thrombocytopenias are a heterogeneous group of disorders characterized by decreased platelet number or function, primarily arising from underlying genetic defects. Many of these conditions involve genes encoding transcription factors, splice factors, or signaling receptors with roles beyond platelet biology, which helps explain the extra-hematological manifestations frequently observed [48]. While MYH9-related disorders, discussed above, represent the prototype disorder within this category, pathogenic variants in *RUNX1*, ANKRD26, and ETV6 have also been identified as causes of inherited thrombocytopenias and are associated with an increased predisposition to hematologic malignancies [49].

Limited data exist regarding pregnancy and delivery management in patients with inherited thrombocytopenias. In a review of 339 pregnancies in 181 women by Noris et al., 13 different forms of inherited thrombocytopenia were described, including Bernard–Soulier syndrome, platelet-type von Willebrand disease, and MYH9-related disorders [42]. The remaining disorders included ANKRD26, ACTN1, RUNX1, and ITGB3-related thrombocytopenias, reported in 21, 9, 4, and 3 patients, respectively. Aside from maintaining a platelet count above 50 × 10^9^/L at the time of delivery, no additional specific management recommendations were proposed. This threshold was based on the observation that women with inherited thrombocytopenias and platelet counts below 50 × 10^9^/L were more likely to experience bleeding and had an increased risk of postpartum hemorrhage. Notably, bleeding risk during pregnancy itself was not increased compared with the general population. The study was not powered, to assess the safest mode of delivery [42].

## 7. Platelet Function Defects

While other inherited platelet disorders have been described, platelet functional defects remain a particularly challenging subgroup to manage during delivery. Patients with platelet functional defects may present with bleeding and nonspecific findings on platelet aggregation testing or variants of uncertain significance identified on genetic testing. Unfortunately, there is no direct evidence guiding the management of platelet functional defects during pregnancy. As a result, management principles are largely extrapolated from other inherited platelet disorders, including the use of transfusions when clinically indicated [50].

## 8. Conclusions

Inherited platelet disorders represent a diverse group of rare conditions that pose distinct challenges during pregnancy, delivery, and the postpartum period. Across disorders, bleeding risk is influenced by underlying platelet dysfunction or thrombocytopenia, the hemostatic demands of childbirth, and the need to balance maternal and neonatal safety. While pregnancy itself does not consistently worsen bleeding phenotype, the peripartum period remains a time of heightened risk, particularly for postpartum hemorrhage.

Management strategies vary substantially by disorder and are largely informed by case series and expert consensus rather than prospective data. A multidisciplinary approach, early delivery planning, and individualized hemostatic strategies are central to optimizing outcomes. Differences between platelet function disorders and inherited thrombocytopenias underscore the importance of tailoring management based on mechanism of disease, prior bleeding history, and response to therapy.

Significant knowledge gaps remain, including limited data on optimal prophylactic strategies, anesthesia management, neonatal outcomes, and the role of emerging therapies. Future efforts should prioritize prospective registries and standardized reporting of pregnancy outcomes in women with inherited platelet disorders to better inform evidence-based care.

## Data Availability

No new data were created or analyzed in this study. Data sharing is not applicable to this article.
